# Dynamic associations among selected healthy dietary behaviors, physical activity, emotion, and fitness performance in adolescents: a four-wave random intercept cross-lagged panel study

**DOI:** 10.3389/fnut.2026.1900491

**Published:** 2026-08-03

**Authors:** Yu Deng, Tong Liu, Ziliang Wen, Meng Zhang

**Affiliations:** 1 School of Physical Education, Shanghai University of Sport, Shanghai, China; 2 High School Affiliated to Southwest University, Chongqing, China

**Keywords:** adolescence, emotion, fitness performance, longitudinal study, physical activity, random intercept cross-lagged panel model, selected healthy dietary behaviors

## Abstract

**Introduction:**

Adolescent diet, physical activity, emotion, and fitness performance may develop together, yet they are often studied separately. This study examined within-person longitudinal associations among selected healthy dietary behaviors, physical activity, emotion, and fitness performance across one academic year.

**Methods:**

Data came from a school-based four-wave longitudinal assessment conducted in March, June, September, and December 2025. The analytic sample included 2,209 junior secondary school adolescents with complete data across all waves (1,166 boys, 52.8%; 1,043 girls, 47.2%). Selected healthy dietary behaviors were assessed using four dietary indicators. Physical activity was measured using seven scored sections of the Physical Activity Questionnaire for Adolescents. Emotion was assessed using three emotional state items, and fitness performance using standardized endurance, standing long jump, and grip strength indicators. Random intercept cross-lagged panel models separated stable between-person differences from within-person changes. Sex-related patterns were examined using sex-stratified models and formal multigroup tests.

**Results:**

The final fully constrained model showed good fit. At the between-person level, random intercept correlations among the four domains were not statistically significant in the complete-case model. At the within-person level, selected healthy dietary behaviors and physical activity showed reciprocal temporal associations, as did physical activity and emotion. Fitness performance was associated with subsequent physical activity, whereas selected healthy dietary behaviors, physical activity, and emotion were not directly associated with subsequent fitness performance. Formal multigroup tests detected no statistically significant sex differences in the main cross-lagged paths.

**Discussion:**

Selected healthy dietary behaviors and physical activity were temporally coupled within adolescents. Physical activity may represent a potential behavioral connector linking selected healthy dietary behaviors with emotional state and functional physical capacity. Because mediation, indirect effects, and causal pathways were not tested, these findings should be interpreted as hypothesis-generating temporal associations rather than demonstrated mechanisms. The findings support integrated adolescent health promotion addressing diet and physical activity while considering emotional experience and fitness-related support.

## 1 Introduction

Unhealthy dietary behavior during adolescence is an important concern for nutrition and public health (1, 2). Adolescence is a period of rapid growth, increasing behavioral autonomy, and greater exposure to changing food environments, which may make adolescents more vulnerable to poor dietary patterns (3, 4). Specific dietary behaviors, including fruit and vegetable intake and lower consumption of sugar-sweetened beverages and fast food, are commonly used as practical indicators of adolescent dietary health, yet adolescent nutrition has often been examined through relatively static associations between diet and health profiles (2, 5). Less is known about how changes in selected healthy dietary behaviors are situated within broader developmental processes and how these links unfold over time. Adolescent nutrition is therefore not only a matter of food intake at one point in time. It also reflects a dynamic behavior that may shift with daily routines, school contexts, psychosocial experiences, and physical development (1, 3). A clearer understanding of these dynamic links may help inform nutrition focused health promotion strategies that better reflect how adolescents develop in real life.

This broader view of adolescent nutrition is consistent with three complementary perspectives. First, health behavior clustering and multiple health behavior change perspectives suggest that diet, physical activity, and sedentary behavior do not operate in isolation, but often form healthy, unhealthy, or mixed patterns across youth populations (6, 7). These patterns may be shaped by shared routines, social contexts, and self regulatory demands rather than by a single behavior specific pathway (8, 9). Second, developmental and biopsychosocial perspectives view adolescence as a period in which biological maturation, psychosocial experience, affective functioning, and health behaviors become increasingly interconnected (10, 11). From this perspective, selected healthy dietary behaviors may be linked with physical activity, emotion, and fitness performance through shared routines, motivation, affective experience, and physical functioning. Third, within-person developmental dynamics suggest that observed associations may reflect either stable between-person differences or time specific changes within the same adolescent. Conventional cross-sectional or between-person longitudinal associations cannot fully distinguish these processes, and may therefore obscure whether changes in one domain are temporally related to later changes in another domain (12, 13). This issue is especially relevant when selected healthy dietary behaviors are considered alongside physical activity and fitness performance, because nutrition and movement behaviors are frequently addressed together in adolescent health promotion, and health behavior clusters have been linked to physical, mental, and fitness related indicators (6, 14).

Among the movement related health indicators that may develop alongside dietary behavior, physical activity has received particular attention. Selected healthy dietary behaviors and physical activity are often examined as paired lifestyle behaviors because they are embedded in everyday routines and are frequently targeted together in adolescent health promotion (14, 15). Reviews of health behavior clustering suggest that diet, physical activity, and sedentary behavior often form healthy, unhealthy, or mixed profiles among young people, although the exact pattern of co-occurrence varies across samples and indicators (6, 7). This evidence supports the view that diet and activity may cluster within a broader health behavior profile, but it does not establish the direction or stability of their association. One plausible pathway is that healthier dietary behavior may support activity participation by contributing to energy availability, perceived health, and more structured daily routines. Conversely, regular physical activity may encourage healthier food choices through greater health awareness, self regulatory practice, or a stronger identity as an active adolescent. These pathways remain partly speculative because most existing evidence has been based on cross-sectional clustering or intervention studies rather than repeated assessments of reciprocal change. A longitudinal within-person perspective is therefore needed to examine whether within-person deviations in one behavior are associated with subsequent deviations in the other, beyond stable between-person differences between adolescents (12, 13).

Emotion is another domain that may help explain how adolescent health behaviors are organized over time. Emotional state can influence motivation, perceived energy, and willingness to engage in daily health behaviors, including physical activity and food choices. At the same time, these behaviors may also contribute to later emotional experience. Physical activity has been associated with better mental health and more positive affective outcomes in children and adolescents, although evidence about causality and direction remains less consistent than in adults (16, 17). More proximal evidence from daily life studies suggests that physical activity is often followed by more positive affect and energy, whereas evidence for affect predicting later physical activity is more mixed (18). Dietary behavior has also been linked with adolescent psychological health, with poorer diet quality or unhealthy dietary patterns generally associated with poorer mental health indicators, although the strength and consistency of these associations vary across study designs and dietary measures (19, 20). These findings suggest that emotion, dietary behavior, and physical activity may be connected, but they do not establish a single direction of influence. Adolescents with better emotional states may be more likely to maintain active routines and make healthier choices. In turn, healthier behaviors may support later emotional adjustment. Because emotion is sensitive to recent experiences and contexts, its links with dietary behavior and physical activity may be better understood as dynamic processes rather than stable background characteristics. Examining emotion together with health behaviors may therefore provide a fuller account of how behavioral and emotional development unfold during adolescence, especially when stable between-person differences are separated from within-person changes over time (12, 13).

Taken together, these lines of evidence suggest that selected healthy dietary behaviors, physical activity, emotion, and fitness performance may be meaningfully examined as interconnected domains of adolescent health development. Selected healthy dietary behaviors and physical activity represent key lifestyle behaviors that often cluster with other health related behaviors in youth populations (6). Emotion reflects a context-sensitive psychological process that may be linked with daily behavior and recent experience, while fitness performance captures functional physical capacity and provides an objective complement to self reported behavior (16, 21). From a developmental perspective, studying these domains in isolation may miss how behavioral, emotional, and physical processes are coordinated during adolescence (10). For example, a change in selected healthy dietary behaviors may be related to later physical activity, but this association may look different when emotional state and fitness performance are considered at the same time. Similarly, physical activity may serve as a potential behavioral link across nutrition related behavior, emotional state, and physical capacity, rather than functioning only as a separate health behavior. An integrated longitudinal model is therefore needed to clarify which associations reflect stable differences between adolescents and which associations reflect changes unfolding within the same adolescent over time (12, 13).

**Figure 1:**
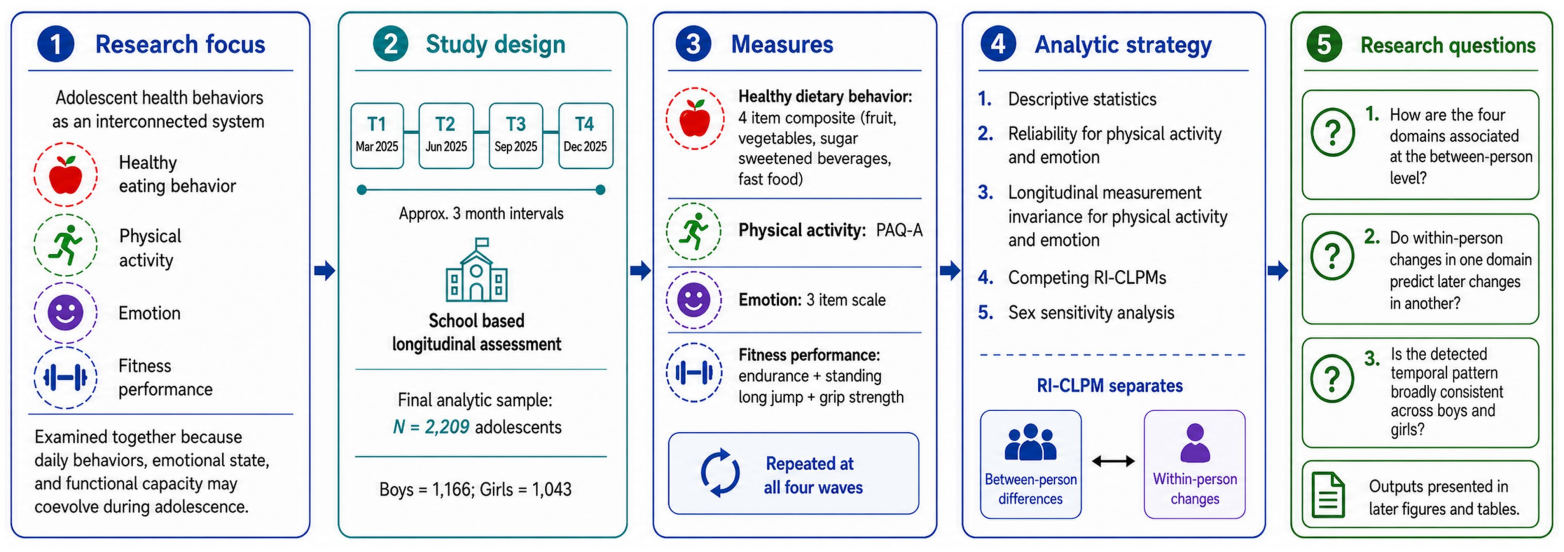
Conceptual and analytic framework overview of the study focus, four wave design, measures, analytic strategy, and research questions. PAQ-A, physical activity questionnaire for adolescents; RI-CLPM, random intercept cross lagged panel model.

Building on this integrated perspective, the present study examined the dynamic associations among selected healthy dietary behaviors, physical activity, emotion, and fitness performance in adolescents using a four wave longitudinal design. To separate stable between-person differences from within-person changes, we applied a random intercept cross-lagged panel model (RI-CLPM) (12, 13). This approach allowed us to examine whether adolescents who generally showed healthier dietary behavior, higher physical activity, better emotional state, or better fitness performance also differed from their peers across the study period. It also allowed us to test whether temporary changes within the same adolescent were associated with subsequent changes in other domains. The conceptual and analytic framework is shown in Figure 1. Rather than proposing directional hypotheses, this study addressed three research questions. First, how are selected healthy dietary behaviors, physical activity, emotion, and fitness performance associated at the between-person level? This question identifies whether adolescents with generally higher levels in one domain also tend to show higher or lower levels in other domains. Second, are within-person changes in these domains associated with later changes in one another over time? This question examines whether a temporary increase or decrease in one domain, such as healthier dietary behavior or higher physical activity than an adolescent’s own usual level, is followed by later changes in another domain, such as emotion or fitness performance. Third, is the selected temporal pattern broadly consistent across boys and girls? This question examines whether the overall pattern can be interpreted as a general adolescent process or whether sex specific interpretation may be needed. Accordingly, any linking role of physical activity was treated as a hypothesis-generating temporal pattern rather than evidence of mediation, indirect effects, or causal pathways.

## 2 Methods

### 2.1 Study design, participants, and sample flow

This study was based on a school-based longitudinal health assessment conducted across one academic year. Four waves of data collection were completed in March, June, September, and December 2025, with an interval of approximately 3 months between adjacent waves. This repeated assessment design allowed us to examine how selected healthy dietary behaviors, physical activity, emotion, and fitness performance were linked over time during adolescence.

The study was conducted in public junior secondary schools in Chongqing, China. All participants were junior secondary school students. Individual chronological age and detailed grade-level information were not retained in the analytic dataset; therefore, the sample is described as junior secondary school adolescents rather than by mean age or specific grade group. This limitation is acknowledged when interpreting the findings.

Data collection was organized at the class level. At each wave, students completed questionnaires on health behavior and emotion, and their fitness performance was assessed through routine school-based fitness testing. Because data collection was embedded in regular school settings, the number of participating students varied across waves. A total of 3,218 students participated at Wave 1 (T1), 3,174 at Wave 2 (T2), 3,391 at Wave 3 (T3), and 3,240 at Wave 4 (T4). These differences mainly reflected absence on specific assessment days, school or class administrative changes, and incomplete questionnaire or fitness-testing records rather than deliberate withdrawal from the target population.

The analytic sample was constructed in two steps. First, students were retained if they had valid data for the core variables across all four waves. Second, records were checked for consistency in school-based identifiers and basic demographic information across waves to ensure that repeated measurements referred to the same student. Fourteen students were excluded because their basic information was inconsistent across waves and could not be reliably matched. The final complete-case analytic sample included 2,209 adolescents, including 1,166 boys and 1,043 girls. Participant flow and sample characteristics are presented in Table 1.

**Table 1:** Participant flow and sex distribution across the four waves.

Assessment/sample stage	Total *n*	Boys, *n* (%)	Girls, *n* (%)	Notes
T1	3,218	1,789 (55.6)	1,429 (44.4)	March 2025
T2	3,174	1,555 (49.0)	1,619 (51.0)	June 2025
T3	3,391	1,716 (50.6)	1,675 (49.4)	September 2025
T4	3,240	1,626 (50.2)	1,614 (49.8)	December 2025
Complete four-wave records before consistency checking	2,223	—	—	Retained before removing inconsistent records
Excluded because of inconsistent basic information	14	—	—	Records could not be reliably matched
Final analytic sample	2,209	1,166 (52.8)	1,043 (47.2)	Included in the main analyses

Percentages were calculated within each row. Differences in sample size across waves mainly reflected student absence during specific assessment rounds. The final analytic sample consisted of junior secondary school adolescents with valid four-wave data for the core study variables after record consistency checking; individual chronological age and detailed grade-level information were not retained in the analytic dataset.

### 2.2 Procedures and ethics

Data were collected through a combination of online and offline procedures. Questionnaire data were obtained using either online or paper-based formats, depending on school arrangements and assessment conditions. For the online component, students completed the questionnaire through Wenjuanxing during scheduled computer classes under unified administration. For the paper-based component, students completed the same questionnaire in classroom settings using standardized instructions. Before each assessment, trained research staff and school personnel explained the purpose of the study, the voluntary nature of participation, and the requirements for completing the questionnaire. Students completed the questionnaires individually under supervision. Research staff were available to answer procedural questions, but they did not influence students’ responses.

Fitness performance data were collected offline through school-based physical fitness assessments. These assessments were conducted by trained personnel following standardized school testing procedures based on the National Student Physical Health Standard (revised in 2014) (Ministry of Education of the People’s Republic of China, 2014). Before formal data collection, the research team held training and coordination meetings to standardize testing procedures, safety requirements, equipment use, scoring criteria, and data-recording procedures across testers and schools.

School-based identifiers and basic demographic information, including school, class, student identifier, and sex, were used only to match student records across the four waves. After record matching and data checking, the dataset was anonymized before analysis.

The study was reviewed and approved by the Institutional Review Board of Shanghai University of Sport. The approval number was 102772025RT092, and approval was granted on March 4, 2025. Written informed consent was obtained from both students and their legal guardians before participation. All procedures were conducted in accordance with ethical standards for research involving human participants.

### 2.3 Measures

The study included four core variables: selected healthy dietary behaviors, physical activity, emotion, and fitness performance. All variables were assessed at four waves. Higher scores indicated a healthier pattern across the selected dietary behaviors, more physical activity, better emotional state, and better fitness performance. The measurement and scoring procedures are summarized in Table 2.

**Table 2:** Summary of measurement and scoring procedures.

Construct	Measurement	Scoring	Indicator type	Reliability	Longitudinal measurement invariance
Selected healthy dietary behaviors	Four items adapted from GSHS: fruit intake, vegetable intake, sugar-sweetened beverage consumption, and fast-food consumption	Five-point scale (1–5); sugar-sweetened beverage and fast-food items reverse-coded; items averaged by wave; a healthier pattern across the selected dietary behaviors	Formative composite	Not estimated	Not estimated
Physical activity	Chinese revised PAQ-A; seven scored section scores	Seven section scores averaged by wave; higher scores indicate higher self-reported physical activity during the previous 7 days	Reflective scale	Estimated	Tested
Emotion	Three items adapted from the AFFEXX core affective exercise experiences component	Three items averaged by wave; higher scores indicate a better emotional state	Reflective scale	Estimated	Tested
Fitness performance	Endurance run, standing long jump, and grip strength based on standardized school fitness testing	Indicators standardized using the T1 reference; endurance time multiplied by −1; indicators averaged by wave; higher scores indicate better fitness performance	Composite performance indicator	Not estimated	Not estimated

GSHS, global school-based student health survey; PAQ-A, physical activity questionnaire for adolescents; AFFEXX, affective exercise experiences questionnaire.

#### 2.3.1 Selected healthy dietary behaviors

Selected healthy dietary behaviors were assessed using four dietary behavior items adapted from the Global School-based Student Health Survey (GSHS). The GSHS is a school-based survey developed by the World Health Organization and the Centers for Disease Control and Prevention to assess health behaviors and protective factors among school-aged adolescents (22). The items used in the present study covered fruit intake, vegetable intake, sugar-sweetened beverage consumption, and fast-food consumption, which are commonly included in GSHS-based assessments of adolescent dietary behavior (23). Full item wording and response categories are provided in Supplementary Table S1.

To fit the assessment format of the present study, the original categorical response format was adapted to a five-point scale. In the final composite, possible scores ranged from 1 to 5. Fruit and vegetable intake were coded in the original direction, whereas sugar-sweetened beverage consumption and fast-food consumption were reverse-coded. The four items were then averaged so that higher scores indicated a healthier pattern across the selected dietary behaviors. This composite was intended to capture selected diet-related behaviors rather than comprehensive diet quality, energy intake, dietary diversity, nutrient adequacy, or meal timing.

Selected healthy dietary behaviors were treated as a formative composite indicator rather than a reflective scale. The four dietary behaviors describe different aspects of adolescents’ eating patterns. They are not expected to be interchangeable manifestations of a single underlying trait. For example, an adolescent may report frequent fruit intake while also reporting frequent sugar-sweetened beverage consumption. This treatment is consistent with the view that composite indicators can combine distinct components that together define an index, rather than assuming that all items are effects of the same latent construct (24). For this reason, internal consistency coefficients and longitudinal measurement invariance were not estimated for selected healthy dietary behaviors.

#### 2.3.2 Physical activity

Physical activity was measured using the Chinese revised version of the Physical Activity Questionnaire for Adolescents (PAQ-A), which was developed and validated for Chinese adolescents (25–27). The PAQ-A is a self-administered 7-day recall instrument designed to assess general physical activity among adolescents during the school year. The questionnaire used in the present study assessed adolescents’ activity participation across typical daily and weekly contexts.

The present study followed the seven-section scoring procedure of the Chinese revised PAQ-A rather than applying an *ad hoc* modification. The first section asked students to report participation in 21 common physical activities and was converted into one section score. Sections 2 to 6 assessed activity in typical school-day and leisure contexts, and the seventh section assessed activity participation across the 7 days of the week. The seven section scores were averaged to calculate the physical activity score at each wave, with higher scores indicating higher self-reported physical activity during the previous 7 days.

Because these sections were designed to reflect a common underlying level of physical activity, internal consistency was evaluated using Cronbach’s alpha and omega at each wave. Reliability was calculated using the seven PAQ-A section scores rather than all individual activity examples as separate scale items. Longitudinal measurement invariance was also tested before the main longitudinal analyses to examine whether the scale structure was comparable across the four waves.

#### 2.3.3 Emotion

Emotion was assessed using three items adapted from the core affective exercise experiences component of the Affective Exercise Experiences (AFFEXX) questionnaire (28). The three items represented pleasure-displeasure, energy-tiredness, and calmness-tension, which are central dimensions of affective experience in physical activity contexts. The abbreviated three-item format was used to reduce respondent burden in the four-wave school-based survey while retaining the core affective dimensions most directly relevant to adolescents’ activity experience.

A mean score was calculated at each wave, with higher scores indicating a better emotional state. Full item wording and response options are provided in Supplementary Table S1, and standardized factor loadings across waves are reported in Supplementary Table S2. Because the items were intended to reflect a common emotional state, internal consistency was evaluated using Cronbach’s alpha and omega at each wave. Longitudinal measurement invariance was tested across the four waves before the longitudinal associations involving emotion were examined.

#### 2.3.4 Fitness performance

Fitness performance was assessed through school-based physical fitness testing following standardized procedures based on the National Student Physical Health Standard (revised in 2014) (Ministry of Education of the People’s Republic of China, 2014). Three indicators were used to represent overall functional physical capacity: endurance, standing long jump, and grip strength. These indicators were selected because they were available in the school-based testing protocol and captured complementary aspects of adolescent fitness, including cardiorespiratory endurance, lower limb explosive strength, and upper body muscular strength. Physical fitness is commonly treated as a marker of functional capacity and youth health, rather than as a self-reported behavior alone (21, 29).

Endurance was assessed using a 1,000 m run for boys and an 800 m run for girls, with completion time recorded in seconds. Because a shorter completion time indicates better performance, endurance time was multiplied by −1 before standardization. Because boys and girls completed different endurance tests, endurance was standardized within sex using the T1 distribution as the reference, and the same T1 standardization parameters were applied to the later waves. Standing long jump was recorded in centimeters, and grip strength was recorded in kilograms. These two indicators were standardized using the overall T1 distribution as the reference, and the same T1 standardization parameters were applied to the later waves. At each wave, the standardized endurance, standing long jump, and grip strength scores were averaged to create a composite fitness performance score.

Fitness performance was treated as a composite performance indicator rather than a reflective scale. The three indicators represent different dimensions of physical capacity and are not expected to be interchangeable items. Therefore, Cronbach’s alpha, omega, and longitudinal measurement invariance were not estimated for fitness performance.

### 2.4 Statistical analysis

All analyses were conducted using R and Mplus. Descriptive statistics were calculated for selected healthy dietary behaviors, physical activity, and emotion at each wave. Fitness performance was not summarized in the descriptive table because it was constructed as a wave-specific standardized composite and was used as a longitudinal model variable. For physical activity and emotion, internal consistency was evaluated using Cronbach’s alpha and omega because these variables were measured as reflective scale-based constructs (30). Internal consistency was not estimated for selected healthy dietary behaviors or fitness performance because both variables were treated as composite indicators rather than reflective scales (24).

Longitudinal measurement invariance was then tested for physical activity and emotion across the four waves. Configural, metric, and scalar invariance models were estimated sequentially, following common measurement invariance testing practice (31). Model fit was evaluated using the comparative fit index (CFI), Tucker-Lewis index (TLI), root mean square error of approximation (RMSEA), and standardized root mean square residual (SRMR). Values of CFI and TLI close to or above 0.90, and values of RMSEA and SRMR close to or below 0.08, were considered to indicate acceptable fit, with higher CFI and TLI and lower RMSEA and SRMR indicating better fit (32). A more constrained invariance model was considered supported when the decrease in fit remained within conventional criteria. These criteria included ΔCFI ≤ 0.010, ΔRMSEA ≤ 0.015, and ΔSRMR ≤ 0.030 for the metric step, and ΔSRMR ≤ 0.010 for the scalar step (33, 34). Selected healthy dietary behaviors and fitness performance were not included in the measurement invariance tests because they were not treated as reflective latent scales.

The primary analyses used the complete-case sample so that model comparison, measurement summaries, and sex-stratified estimates were based on the same four-wave analytic sample. To evaluate potential attrition bias, adolescents included in the complete-case analytic sample were compared with those excluded because of incomplete four-wave data on baseline sex and T1 study variables. Standardized mean differences were reported alongside *p* values because trivial differences may become statistically significant in large samples. As a sensitivity analysis, the RI-CLPM was re-estimated using full information maximum likelihood (FIML) among adolescents with at least one wave of available data. This sensitivity analysis evaluated whether the main within-person pattern was robust when all available longitudinal information was used under the missing-at-random assumption. Because individual reasons for missingness were not recorded for every missing entry, missingness could not be assumed to be completely at random.

The main longitudinal analyses were conducted using RI-CLPMs. This approach separates stable between-person differences from within-person fluctuations over time (12, 13). Random intercepts were specified for selected healthy dietary behaviors, physical activity, emotion, and fitness performance to capture stable differences between adolescents. Within-person latent variables were then used to estimate autoregressive and cross-lagged paths among the four domains across adjacent waves. This allowed us to examine whether short-term deviations from an adolescent’s usual level in one domain were associated with subsequent deviations in another domain. Standardized coefficients were reported directly, and fixed qualitative labels such as small, medium, or large were not used as formal interpretive thresholds.

To identify a parsimonious longitudinal structure, four competing RI-CLPMs were estimated. The baseline model freely estimated autoregressive and cross-lagged paths across time. The autoregressive-constrained model constrained corresponding autoregressive paths to be equal across adjacent intervals. The cross-lagged-constrained model constrained corresponding cross-lagged paths to be equal across adjacent intervals. The fully constrained model constrained both autoregressive and cross-lagged paths to equality over time. Because the four waves were equally spaced at approximately three-month intervals, equality constraints were tested as an empirical stationarity assumption rather than imposed *a priori*. The final model was selected according to overall fit, likelihood-based model comparison where available, and parsimony. When a more constrained model did not show meaningful deterioration in fit relative to the baseline model, the more parsimonious model was retained.

Given the number of estimated temporal paths, Benjamini-Hochberg false discovery rate correction was applied to the 36 adjacent cross-lagged paths as a sensitivity check for multiple testing (35). The corrected pattern was summarized in the Results.

Sex-related patterns were examined in two steps. First, the final RI-CLPM was estimated separately among boys and girls to describe whether the selected model structure showed acceptable fit in both groups. Second, formal multigroup RI-CLPM tests were conducted to evaluate whether the dynamic pathways differed by sex. Joint Wald tests were used to test equality of the 12 adjacent cross-lagged paths and a broader set of 16 dynamic parameters that included autoregressive and cross-lagged paths. Sex-stratified estimates were therefore interpreted as descriptive unless supported by formal multigroup evidence.

## 3 Results

### 3.1 Descriptive statistics and scale properties

Descriptive statistics and reliability estimates are presented in Table 3. The final analytic sample included 2,209 junior secondary school adolescents with complete data for the core variables across all waves. Mean scores for selected healthy dietary behaviors showed modest wave-to-wave variation, ranging from 3.39 to 3.59. Physical activity scores ranged from 3.06 to 3.15, and emotion scores ranged from 2.93 to 2.97, indicating broadly stable average levels across the study period. Fitness performance was not included in Table 3 because it was constructed as a wave-specific standardized composite and had limited descriptive value as a raw mean score.

**Table 3:** Descriptive statistics and reliability estimates across four waves (*n* = 2,209).

Variable	Wave	Mean (±SD)	Cronbach’s alpha	Omega
Selected healthy dietary behaviors	T1	3.59 (±1.22)	—	—
	T2	3.52 (±1.21)	—	—
	T3	3.39 (±1.21)	—	—
	T4	3.48 (±1.22)	—	—
Physical activity	T1	3.06 (±1.36)	0.988	0.988
	T2	3.15 (±1.53)	0.997	0.997
	T3	3.06 (±1.56)	0.989	0.989
	T4	3.09 (±1.54)	0.976	0.977
Emotion	T1	2.93 (±0.99)	0.920	0.920
	T2	2.94 (±0.99)	0.976	0.976
	T3	2.97 (±1.02)	0.947	0.947
	T4	2.94 (±1.02)	0.921	0.921

Reliability estimates were reported only for reflective scale-based constructs. Selected healthy dietary behaviors were treated as a formative composite indicator; therefore, Cronbach’s alpha and omega were not estimated. Physical activity reliability estimates were based on the seven PAQ-A section scores rather than all individual activity examples as separate items. Fitness performance is not shown because it was constructed from T1-referenced standardized performance indicators and was used only as a composite score in the longitudinal model.

Reliability estimates were reported only for the reflective scale-based constructs. For physical activity, Cronbach’s alpha ranged from 0.976 to 0.997, and omega ranged from 0.977 to 0.997 across the four waves. These estimates were based on the seven PAQ-A section scores rather than all activity examples as separate items. For emotion, Cronbach’s alpha ranged from 0.920 to 0.976, and omega ranged from 0.920 to 0.976.

Attrition analyses compared adolescents included in the complete-case analytic sample (*n* = 2,209) with those excluded because of incomplete four-wave data (*n* = 1,546). Baseline differences were small. All absolute standardized mean differences were below 0.10 for sex, T1 selected healthy dietary behaviors, T1 physical activity, T1 emotion, and T1 fitness performance. Although the baseline difference in selected healthy dietary behaviors reached statistical significance (*p* = 0.011), its standardized mean difference was small (SMD = 0.090), indicating negligible baseline imbalance.

### 3.2 Longitudinal measurement invariance

Longitudinal measurement invariance was tested for physical activity and emotion across the four waves. The results are shown in Table 4. For physical activity, the configural model showed good fit to the data, *χ*^2^ = 380.823, *df* = 302, CFI = 0.999, TLI = 0.999, RMSEA = 0.011, and SRMR = 0.007. The metric model also showed good fit, and changes from the configural model were negligible. The scalar model remained acceptable, *χ*^2^ = 762.365, *df* = 338, CFI = 0.994, TLI = 0.993, RMSEA = 0.024, and SRMR = 0.009. Compared with the metric model, changes in fit were within the predefined criteria, with ΔCFI = 0.005, ΔRMSEA = 0.012, and ΔSRMR = 0.001. These results supported scalar longitudinal measurement invariance for physical activity.

**Table 4:** Longitudinal measurement invariance across four waves.

Model	*χ*^2^(*df*)	CFI	TLI	SRMR	RMSEA [90% CI]	ΔRMSEA	ΔCFI	ΔSRMR
Physical activity								
Configural invariance	380.823 (302)	0.999	0.999	0.007	0.011 [0.007, 0.014]			
Metric invariance	418.819 (320)	0.999	0.998	0.008	0.012 [0.008, 0.015]	0.001	0.000	0.001
Scalar invariance	762.365 (338)	0.994	0.993	0.009	0.024 [0.022, 0.026]	0.012	0.005	0.001
Emotion								
Configural invariance	27.869 (30)	1.000	1.000	0.004	0.000 [0.000, 0.015]			
Metric invariance	31.984 (36)	1.000	1.000	0.006	0.000 [0.000, 0.013]	0.000	0.000	0.002
Scalar invariance	36.725 (42)	1.000	1.000	0.007	0.000 [0.000, 0.011]	0.000	0.000	0.001

Δ values refer to changes relative to the previous model. Metric invariance was compared with configural invariance, and scalar invariance was compared with metric invariance. Scalar longitudinal measurement invariance was supported for both physical activity and emotion.

For emotion, the configural, metric, and scalar models all showed good fit. The scalar model showed excellent fit, *χ*^2^ = 36.725, *df* = 42, CFI = 1.000, TLI = 1.000, RMSEA = 0.000, and SRMR = 0.007. Changes in fit across successive models were negligible. At the scalar step, ΔCFI = 0.000, ΔRMSEA = 0.000, and ΔSRMR = 0.001. These results supported scalar longitudinal measurement invariance for emotion. Overall, both reflective constructs showed sufficient longitudinal comparability for the subsequent RI-CLPM analyses.

### 3.3 RI-CLPM model comparison and final model results

Model comparison results for the RI-CLPMs are presented in Table 5. The baseline model showed good fit to the data, *χ*^2^ = 117.895, *df* = 38, CFI = 0.998, TLI = 0.993, RMSEA = 0.031, and SRMR = 0.013. The autoregressive constrained model, cross-lagged constrained model, and fully constrained model also showed acceptable fit. Compared with the baseline model, the fully constrained model showed only a small decrease in fit, with ΔCFI = 0.003, ΔRMSEA = 0.003, and ΔSRMR = 0.013. Because these changes were within the predefined criteria and the model provided the most parsimonious representation of the longitudinal structure, the fully constrained model was retained as the final model, *χ*^2^ = 251.411, *df* = 70, CFI = 0.995, TLI = 0.992, RMSEA = 0.034, and SRMR = 0.026.

**Table 5:** Fit indices for competing RI-CLPMs.

Model	*χ*^2^(df)	CFI	TLI	SRMR	RMSEA [90% CI]	ΔRMSEA	ΔCFI	ΔSRMR	BIC
M0: baseline RI-CLPM	117.895 (38)	0.998	0.993	0.013	0.031 [0.025, 0.037]				63034.489
M1: autoregressive constrained RI-CLPM	231.564 (46)	0.995	0.987	0.026	0.043 [0.037, 0.048]	0.012	0.003	0.013	63071.874
M2: cross-lagged constrained RI-CLPM	146.199 (62)	0.998	0.996	0.014	0.025 [0.020, 0.030]	0.006	0.000	0.001	62882.606
M3: fully constrained RI-CLPM	251.411 (70)	0.995	0.992	0.026	0.034 [0.030, 0.039]	0.003	0.003	0.013	62918.925

Δ values refer to changes relative to the baseline RI-CLPM. The fully constrained model was retained as the final model because model fit remained acceptable and deterioration in fit was within the predefined criteria. Equality constraints were treated as an empirical stationarity assumption because waves were equally spaced at approximately three-month intervals.

**Figure 2:**
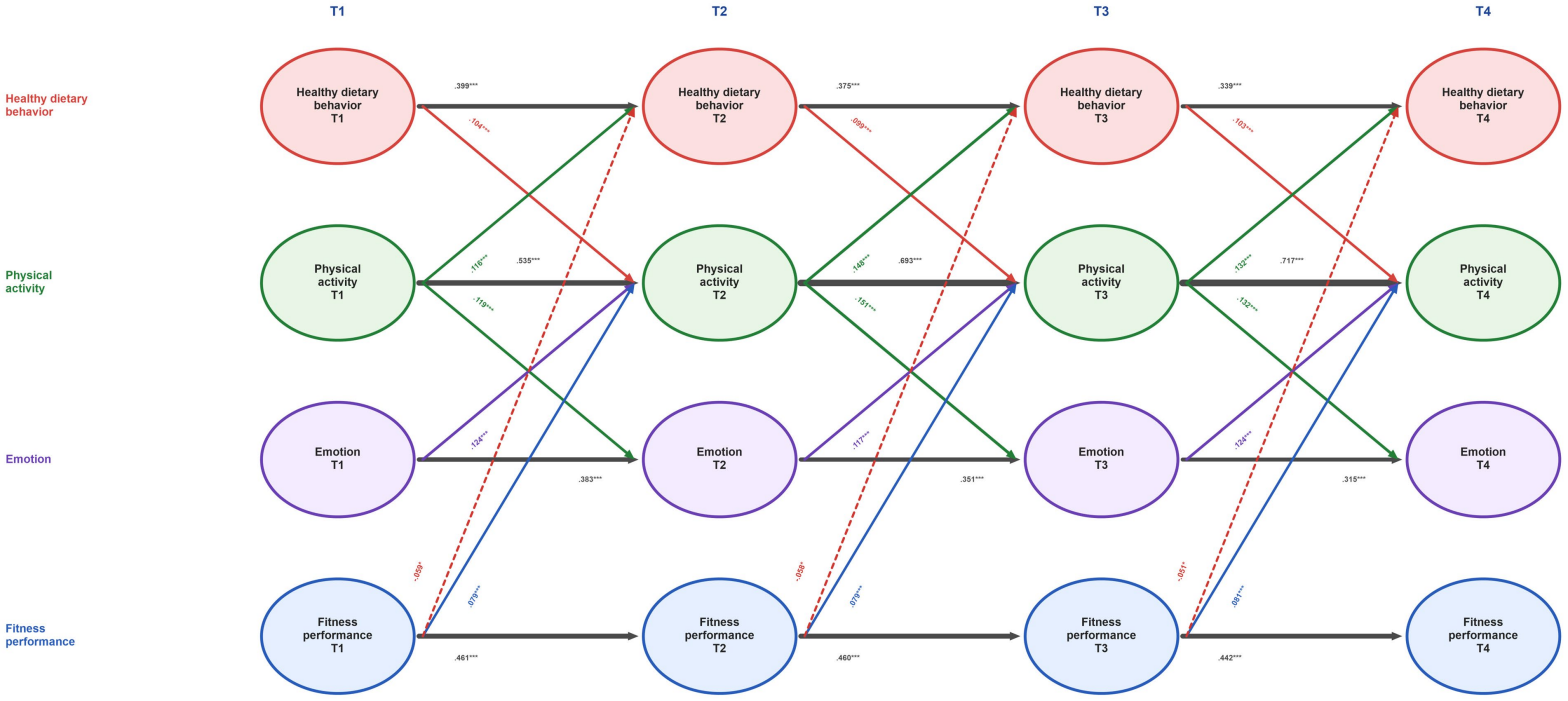
Simplified RI-CLPM results: Standardized estimates from the final model are shown. Black arrows indicate autoregressive paths, colored arrows indicate significant cross-lagged paths, and dashed arrows indicate negative paths. Non-significant paths, random intercepts, and between-person correlations are omitted for clarity. Significance markers refer to the final complete-case model before the FDR sensitivity correction. RI-CLPM, random intercept cross-lagged panel model. *p < 0.05, **p < 0.01, ***p < 0.001.

Standardized estimates from the final model are shown in Table 6, and the simplified model is presented in Figure 2. Because equality constraints were imposed on unstandardized estimates, standardized coefficients could vary slightly across intervals due to wave-specific variances. At the between-person level in the complete-case model, none of the random intercept correlations among selected healthy dietary behaviors, physical activity, emotion, and fitness performance were statistically significant. At the within-person level, all four variables showed significant autoregressive effects across adjacent waves. The autoregressive coefficients ranged from 0.339 to 0.399 for selected healthy dietary behaviors, from 0.535 to 0.717 for physical activity, from 0.315 to 0.383 for emotion, and from 0.442 to 0.461 for fitness performance, with all *p* values < 0.001. These results indicated that deviations from adolescents’ own usual levels tended to carry forward to the next wave. The strongest within-person stability was observed for physical activity.

**Table 6:** Standardized path estimates from the final RI-CLPM.

Path	T1 → T2 *β* (SE)	T2 → T3 *β* (SE)	T3 → T4 β (SE)
Autoregressive paths			
Selected healthy dietary behaviors → Selected healthy dietary behaviors	0.399*** (0.030)	0.375*** (0.035)	0.339*** (0.037)
Physical activity → physical activity	0.535*** (0.020)	0.693*** (0.023)	0.717*** (0.024)
Emotion → emotion	0.383*** (0.031)	0.351*** (0.042)	0.315*** (0.038)
Fitness performance → fitness performance	0.461*** (0.027)	0.460*** (0.037)	0.442*** (0.041)
Cross-lagged paths			
Selected healthy dietary behaviors → physical activity	0.104*** (0.022)	0.099*** (0.022)	0.103*** (0.023)
Physical activity → Selected healthy dietary behaviors	0.116*** (0.026)	0.148*** (0.033)	0.132*** (0.029)
Selected healthy dietary behaviors → emotion	−0.007 (0.030)	−0.006 (0.028)	−0.006 (0.025)
Emotion → selected healthy dietary behaviors	0.026 (0.028)	0.025 (0.026)	0.022 (0.024)
Selected healthy dietary behaviors → fitness performance	−0.038 (0.027)	−0.036 (0.026)	−0.035 (0.025)
Fitness performance → selected healthy dietary behaviors	−0.059* (0.028)	−0.058* (0.028)	−0.051* (0.025)
Physical activity → emotion	0.119*** (0.028)	0.151*** (0.035)	0.132*** (0.030)
Emotion → physical activity	0.124*** (0.023)	0.117*** (0.022)	0.124*** (0.023)
Physical activity → fitness performance	−0.010 (0.025)	−0.014 (0.033)	−0.013 (0.032)
Fitness performance → physical activity	0.079*** (0.022)	0.079*** (0.021)	0.081*** (0.021)
Emotion → fitness performance	0.010 (0.027)	0.010 (0.025)	0.010 (0.025)
Fitness performance → emotion	0.043 (0.033)	0.042 (0.032)	0.037 (0.028)

Values are standardized estimates with standard errors in parentheses. Qualitative small/medium/large labels were not applied as formal interpretive thresholds. *p < 0.05, **p < 0.01, ***p < 0.001.

Several cross-lagged paths were statistically significant. Higher-than-usual selected healthy dietary behaviors were associated with subsequent higher physical activity across all intervals, beta = 0.099 to 0.104. Physical activity was also associated with subsequent selected healthy dietary behaviors, beta = 0.116 to 0.148. Physical activity and emotion showed reciprocal temporal associations. Physical activity was associated with subsequent emotion, beta = 0.119 to 0.151, and emotion was associated with subsequent physical activity, beta = 0.117 to 0.124. Fitness performance was associated with subsequent physical activity, beta = 0.079 to 0.081. Fitness performance also showed a weak negative association with subsequent selected healthy dietary behaviors, beta = −0.059 to −0.051, *p* = 0.036 to 0.044. Other cross-lagged paths were not statistically significant. These included emotion to selected healthy dietary behaviors, selected healthy dietary behaviors to emotion, fitness performance to emotion, selected healthy dietary behaviors to fitness performance, physical activity to fitness performance, and emotion to fitness performance.

Multiple-testing sensitivity analyses using Benjamini-Hochberg false discovery rate correction were applied to the 36 adjacent cross-lagged paths. The main pattern was unchanged: the reciprocal temporal associations between selected healthy dietary behaviors and physical activity, the reciprocal temporal associations between physical activity and emotion, and the paths from fitness performance to physical activity remained significant after correction. The weak negative paths from fitness performance to selected healthy dietary behaviors did not remain significant after FDR correction and were therefore treated as less robust.

The FIML sensitivity model based on all available participants with at least one wave of core longitudinal data (*n* = 3,416) also showed good fit, chi-square = 348.009, *df* = 70, CFI = 0.995, TLI = 0.991, RMSEA = 0.034, and SRMR = 0.024. The core within-person pattern was substantively consistent with the complete-case model, including reciprocal temporal associations between selected healthy dietary behaviors and physical activity, reciprocal temporal associations between physical activity and emotion, and positive paths from fitness performance to subsequent physical activity. At the between-person level, associations remained generally weak, although the random intercept correlation between physical activity and emotion reached statistical significance in the FIML model.

### 3.4 Sex-stratified and multigroup analyses

The final fully constrained RI-CLPM was first repeated separately among boys and girls. The model showed acceptable fit in both groups. For boys, model fit was chi-square = 194.929, *df* = 70, CFI = 0.994, TLI = 0.989, RMSEA = 0.039, and SRMR = 0.027. For girls, model fit was chi-square = 154.092, *df* = 70, CFI = 0.995, TLI = 0.992, RMSEA = 0.034, and SRMR = 0.028. These results indicated that the selected model structure was broadly acceptable in both sex groups (Table 7).

**Table 7:** Sex-stratified and multigroup RI-CLPM analyses.

Test	n	Result
Boys	1,166	*χ*^2^(70) = 194.929, CFI = 0.994, TLI = 0.989, SRMR = 0.027, RMSEA = 0.039 [0.032, 0.045]
Girls	1,043	*χ*^2^(70) = 154.092, CFI = 0.995, TLI = 0.992, SRMR = 0.028, RMSEA = 0.034 [0.027, 0.041]
12 cross-lagged paths	—	Wald *χ*^2^(12) = 8.661, p = 0.732
16 dynamic paths	—	Wald *χ*^2^(16) = 13.166, *p* = 0.661

Sex-stratified models examined model fit separately in boys and girls. Formal multigroup Wald tests did not detect statistically significant sex differences in the main dynamic pathways; therefore, sex-stratified estimates were interpreted descriptively.

Formal multigroup RI-CLPM tests were then conducted to evaluate whether the dynamic paths differed by sex. The joint Wald test for the 12 adjacent cross-lagged path differences was not statistically significant, Wald chi-square(12) = 8.661, *p* = 0.732. A broader joint test of 16 dynamic path differences, including autoregressive and cross-lagged paths, was also not significant, Wald chi-square(16) = 13.166, *p* = 0.661. These results did not provide statistical evidence for sex differences in the main dynamic pathways.

**Figure 3:**
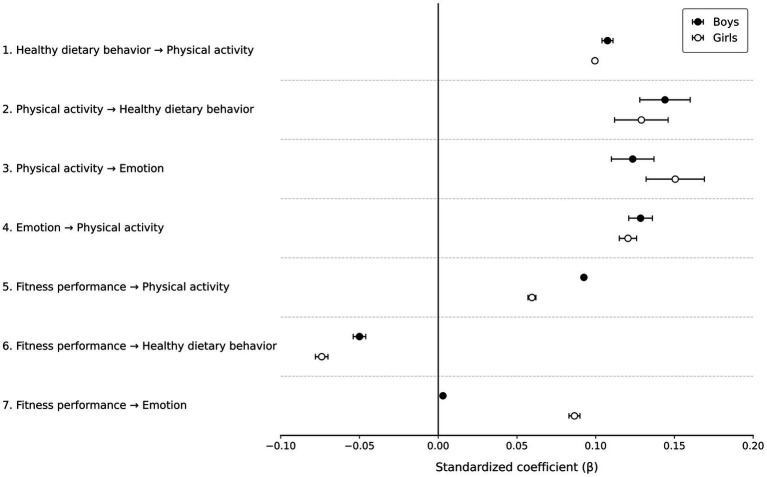
Sex-stratified RI-CLPM estimates. Standardized estimates from the final RI-CLPM are shown separately for boys and girls. Points represent the mean standardized coefficient across the three adjacent-wave intervals, and horizontal bars represent the range across intervals. Only the main cross-lagged paths are displayed. Formal multigroup tests did not detect statistically significant sex differences; therefore, sex-stratified estimates should be interpreted descriptively. RI-CLPM, random intercept cross-lagged panel model.

The sex-stratified estimates were therefore interpreted descriptively (Figure 3). In both groups, selected healthy dietary behaviors were associated with subsequent physical activity, beta = 0.104 to 0.111 in boys and beta = 0.098 to 0.101 in girls. Physical activity was also associated with subsequent selected healthy dietary behaviors, beta = 0.128 to 0.160 in boys and beta = 0.112 to 0.146 in girls. The reciprocal temporal associations between physical activity and emotion were also observed in both groups. Physical activity was associated with subsequent emotion, beta = 0.110 to 0.137 in boys and beta = 0.132 to 0.169 in girls. Emotion was associated with subsequent physical activity, beta = 0.121 to 0.136 in boys and beta = 0.115 to 0.126 in girls. Fitness performance was associated with subsequent physical activity in both groups, beta = 0.092 to 0.093 in boys and beta = 0.057 to 0.062 in girls. Although fitness performance was associated with subsequent emotion in girls, beta = 0.083 to 0.090, this path was not significant in boys, beta = 0.003. Given the nonsignificant multigroup tests, these sex-stratified differences should be interpreted as descriptive variation rather than confirmatory evidence of sex-specific dynamics.

## 4 Discussion

### 4.1 Principal findings

This four-wave longitudinal study examined how selected healthy dietary behaviors, physical activity, emotion, and fitness performance were temporally connected within adolescents across one academic year. After separating stable between-person differences from within-person deviations using an RI-CLPM, the clearer pattern appeared at the within-person level. Selected healthy dietary behaviors and physical activity showed reciprocal temporal associations, and physical activity and emotion also showed reciprocal temporal associations. Fitness performance was associated with subsequent physical activity, whereas selected healthy dietary behaviors, physical activity, and emotion were not directly associated with subsequent fitness performance. These findings should be interpreted as temporal within-person associations rather than evidence of causal effects.

The additional sensitivity analyses strengthened, but also refined, the interpretation of the findings. Attrition analyses showed negligible baseline imbalance between included and excluded participants, and the FIML sensitivity model based on all available longitudinal data produced a substantively similar within-person pattern. Formal multigroup RI-CLPM tests did not detect statistically significant sex differences in the main dynamic pathways. Therefore, the sex-stratified estimates are best viewed as descriptive information rather than evidence of sex-specific mechanisms.

Overall, the results suggest that physical activity may represent a potential behavioral connector within the system examined here. This interpretation remains deliberately cautious. The study did not test mediation, indirect effects, or experimental causal pathways. Physical activity should therefore be understood as a candidate linking behavior suggested by the observed temporal associations, not as a demonstrated mechanism connecting diet, emotion, and fitness performance.

### 4.2 Between-person associations and within-person dynamics

The between-person findings were generally weak. In the complete-case model, random intercept correlations among selected healthy dietary behaviors, physical activity, emotion, and fitness performance were not statistically significant. In the FIML sensitivity model, the between-person association between physical activity and emotion reached statistical significance, but the overall pattern still suggested weaker and less consistent between-person coupling than within-person temporal coupling. Thus, the findings should not be interpreted as showing that the four domains are unrelated at the population level; rather, they indicate that the most consistent links in this dataset were observed when adolescents were compared with their own usual levels over time.

Several factors may explain the relatively weak between-person pattern. The dietary composite captured selected behaviors rather than comprehensive diet quality. Emotion was measured using an abbreviated three-item indicator. Complete-case selection may have reduced between-person variability, although the attrition analysis suggested negligible baseline imbalance. Developmental heterogeneity within adolescence, school context, and restricted variability in a school-based sample may also have limited stable between-person correlations. These considerations support a balanced interpretation of the random intercept results.

The within-person results provide a complementary perspective. They suggest that temporary deviations from an adolescent’s own usual level in one domain were associated with later deviations in another domain. This distinction matters because between-person and within-person associations can reflect different substantive processes and should not be treated as interchangeable (12, 13). For adolescent health promotion, this means that changes occurring within the same student over time may be as informative as stable differences between students.

### 4.3 Selected healthy dietary behaviors and physical activity

The reciprocal temporal association between selected healthy dietary behaviors and physical activity was one of the clearest findings. When adolescents scored higher than usual on selected healthy dietary behaviors, they tended to report higher physical activity at the next wave. Conversely, higher-than-usual physical activity was associated with higher scores on selected healthy dietary behaviors at the next wave. This pattern is consistent with health behavior clustering and multiple health behavior change perspectives, which suggest that diet and physical activity can be shaped by shared routines, social contexts, and self-regulatory demands (6, 8, 9).

This finding should nevertheless be interpreted within the measurement limits of the dietary composite. The four dietary indicators captured fruit intake, vegetable intake, sugar-sweetened beverage consumption, and fast-food consumption, but they did not assess energy intake, breakfast, meal timing, dietary diversity, whole grains, dairy, protein sources, ultra-processed foods, or nutrient adequacy. The term selected healthy dietary behaviors is therefore more precise than overall diet quality. Future studies using more comprehensive dietary assessment could test whether the observed reciprocal pattern extends to broader indices of adolescent diet quality.

### 4.4 Physical activity and emotion

Physical activity and emotion also showed reciprocal temporal associations. Being more active than usual was associated with better subsequent emotional state, and better-than-usual emotional state was associated with higher subsequent physical activity. This pattern is compatible with affective and developmental perspectives suggesting that activity experiences may contribute to enjoyment, energy, social connection, perceived competence, and stress relief, while more positive emotional states may increase adolescents’ willingness to participate in activity (16, 18, 28).

The absence of direct temporal associations between selected healthy dietary behaviors and emotion should also be interpreted cautiously. It does not imply that nutrition is irrelevant to emotional development. Rather, the specific dietary composite used here may have been too broad to capture dietary features that are more proximal to emotional experience, such as meal regularity, total energy intake, emotional eating, nutrient adequacy, or dietary pattern quality. In addition, the emotion measure was an abbreviated adaptation of the AFFEXX core affective experience items rather than the full AFFEXX questionnaire. These measurement choices reduced participant burden in the four-wave school-based survey but also limit the breadth of interpretation.

### 4.5 Fitness performance and physical activity

Fitness performance had a more specific role. Better-than-usual fitness performance was associated with higher subsequent physical activity, whereas physical activity was not directly associated with subsequent fitness performance. This may indicate that adolescents with better functional capacity find activity participation more manageable or rewarding. At the same time, broad self-reported activity may be insufficient to produce measurable short-term change in endurance, standing long jump, and grip strength across a three-month interval. Fitness development is also influenced by biological maturation, body composition, training quality, and school physical education context.

The weak negative path from fitness performance to selected healthy dietary behaviors requires particular caution. Although this path reached nominal significance in the complete-case model, it did not remain significant after Benjamini-Hochberg FDR correction and was not robust in the FIML sensitivity analysis. Therefore, it should not be interpreted as evidence that better fitness leads to poorer dietary behavior. The finding is better treated as a small and uncertain association that requires replication with more detailed dietary and training data.

The sex-stratified estimates suggested some descriptive variation in paths involving fitness performance, but formal multigroup RI-CLPM tests did not support statistically significant sex differences in the dynamic pathways. Sex-specific patterns involving fitness performance may be useful for generating future hypotheses, but they should not be interpreted as confirmed sex-specific processes in the present study.

### 4.6 Integrated interpretation and practical implications

The integrated pattern suggests that adolescent nutrition-related behavior may be better understood within the daily routines and activity contexts in which students make health-related choices. Physical activity was temporally linked with selected healthy dietary behaviors and emotion, and it was also preceded by fitness performance. These findings support considering physical activity as a practical entry point for integrated health promotion, while avoiding the stronger claim that it has been proven to mediate or causally bridge the other domains.

For school-based practice, this interpretation aligns with a whole-school health-promoting approach. The WHO and UNESCO health-promoting schools framework emphasizes coordinated action across school environments, routines, policies, curricula, health services, and community engagement rather than isolated health education alone (36). From this perspective, nutrition-focused interventions may be strengthened when they are integrated with supportive physical activity opportunities and emotionally positive activity experiences.

The reciprocal association between physical activity and emotion also suggests that increasing activity volume alone may be insufficient. Activity programs should aim to create enjoyable, supportive, and competence-building experiences, especially for students with lower fitness performance who may experience activity as more difficult or stigmatizing. Such an approach may help connect nutrition education, activity opportunities, emotional support, and fitness-related support within a coherent school health strategy.

### 4.7 Strengths and limitations

This study has several strengths. The four-wave longitudinal design allowed temporal associations to be examined across one academic year. The RI-CLPM separated stable between-person differences from within-person deviations, providing a clearer view of how changes within the same adolescent were related over time. The study integrated selected healthy dietary behaviors, physical activity, emotion, and fitness performance in one model, and longitudinal measurement invariance was tested for the reflective constructs before the main longitudinal analyses. Robustness was further evaluated through attrition, FIML, FDR, and multigroup sensitivity analyses

Several limitations should be considered. First, although attrition analyses showed negligible baseline imbalance and the FIML sensitivity model yielded a similar within-person pattern, the primary analysis was based on complete cases and selection bias cannot be fully ruled out. Second, the dietary composite captured only selected healthy dietary behaviors and should not be interpreted as comprehensive diet quality. Third, the emotion measure was an abbreviated adaptation of AFFEXX core affective items, which limited the breadth of emotional assessment. Fourth, physical activity was self-reported and no objective activity monitoring was available.

Additional limitations concern unmeasured developmental and contextual factors. Individual chronological age and detailed grade-level information were not retained in the analytic dataset. BMI, pubertal stage, socioeconomic status, parental education, sleep, sedentary behavior, body composition, training history, and school-level contextual variables were not included in the RI-CLPM. These omissions leave residual confounding possible and limit the ability to interpret the temporal paths as causal effects. The follow-up period covered approximately one academic year, and longer follow-up may be needed to understand how these domains co-develop across childhood and adolescence.

Finally, although RI-CLPM improves the separation of within-person and between-person components, it does not establish causality. The findings should therefore be interpreted as longitudinal temporal associations. Future studies should combine more comprehensive dietary measures, objective activity monitoring, biological maturation indicators, contextual covariates, and preregistered longitudinal multigroup models to clarify how dietary behavior, physical activity, emotion, and fitness performance develop together during adolescence.

## 5 Conclusion

This study examined within-person temporal associations among selected healthy dietary behaviors, physical activity, emotion, and fitness performance across one academic year in junior secondary school adolescents. The RI-CLPM results indicated that the most consistent associations were expressed as temporal within-person deviations rather than stable between-person correlations. Selected healthy dietary behaviors and physical activity showed reciprocal temporal associations, and physical activity and emotion also showed reciprocal temporal associations. Fitness performance was associated with subsequent physical activity, whereas selected healthy dietary behaviors, physical activity, and emotion were not directly associated with subsequent fitness performance.

These findings suggest that physical activity may represent a potential behavioral connector within the system examined here, linking selected healthy dietary behaviors with emotional state and functional physical capacity. Because mediation, indirect effects, and causal pathways were not tested, the findings should be interpreted as longitudinal temporal associations rather than demonstrated mechanisms. Nutrition-focused adolescent health promotion may therefore benefit from integrated strategies that combine dietary support with structured, enjoyable, and emotionally supportive physical activity opportunities. Future studies using more detailed dietary assessment, objective activity monitoring, longer follow-up, and broader developmental and contextual covariates are needed to verify and extend these patterns.

## Data Availability

The datasets for this article are not publicly available due to concerns regarding the anonymity and privacy of minor participants. Requests to access the datasets should be directed to the corresponding author, Meng Zhang (2311111011@sus.edu.cn), and will be considered in accordance with applicable ethical, institutional, and data protection requirements.

## References

[1] Hargreaves D Mates E Menon P Alderman H Devakumar D Fawzi W . Strategies and interventions for healthy adolescent growth, nutrition, and development. Lancet. (2022) 399:198–210. doi: 10.1016/S0140-6736(21)01593-2, 34856192

[2] Norris SA Frongillo EA Black MM Dong Y Fall C Lampl M . Nutrition in adolescent growth and development. Lancet. (2022) 399:172–84. doi: 10.1016/S0140-6736(21)01590-7, 34856190

[3] Neufeld LM Andrade EB Ballonoff Suleiman A Barker M Beal T Blum LS . Food choice in transition: adolescent autonomy, agency, and the food environment. Lancet. (2022) 399:185–97. doi: 10.1016/S0140-6736(21)01687-1, 34856191

[4] Sawyer SM Azzopardi PS Wickremarathne D Patton GC. The age of adolescence. Lancet Child Adolesc Health. (2018) 2:223–8. doi: 10.1016/S2352-4642(18)30022-1, 30169257

[5] Patton GC Sawyer SM Santelli JS Ross DA Afifi R Allen NB . Our future: a lancet commission on adolescent health and wellbeing. Lancet. (2016) 387:2423–78. doi: 10.1016/S0140-6736(16)00579-1, 27174304 PMC5832967

[6] Alosaimi N Sherar LB Griffiths P Pearson N. Clustering of diet, physical activity and sedentary behaviour and related physical and mental health outcomes: a systematic review. BMC Public Health. (2023) 23:1572. doi: 10.1186/s12889-023-16372-6, 37596591 PMC10436445

[7] Blyth F Haycraft E Peral-Suarez A Pearson N. Tracking and changes in the clustering of physical activity, sedentary behavior, diet, and sleep across childhood and adolescence: a systematic review. Obes Rev. (2025) 26:e13909. doi: 10.1111/obr.13909, 39967026 PMC12137043

[8] Prochaska JJ Spring B Nigg CR. Multiple health behavior change research: an introduction and overview. Prev Med. (2008) 46:181–8. doi: 10.1016/j.ypmed.2008.02.001, 18319098 PMC2288583

[9] Spring B Champion KE Acabchuk RL Hennessy EA. Self-regulatory behaviour change techniques in interventions to promote healthy eating, physical activity, or weight loss: a meta-review. Health Psychol Rev. (2021) 15:508–39. doi: 10.1080/17437199.2020.1721310, 31973666 PMC7429262

[10] Dahl RE Allen NB Wilbrecht L Suleiman AB. Importance of investing in adolescence from a developmental science perspective. Nature. (2018) 554:441–50. doi: 10.1038/nature25770, 29469094

[11] Sawyer SM Afifi RA Bearinger LH Blakemore SJ Dick B Ezeh AC . Adolescence: a foundation for future health. Lancet. (2012) 379:1630–40. doi: 10.1016/S0140-6736(12)60072-5, 22538178

[12] Hamaker EL Kuiper RM Grasman RPPP. A critique of the cross-lagged panel model. Psychol Methods. (2015) 20:102–16. doi: 10.1037/a0038889, 25822208

[13] Mulder JD Hamaker EL. Three extensions of the random intercept cross-lagged panel model. Struct Equ Model Multidiscip J. (2021) 28:638–48. doi: 10.1080/10705511.2020.1784738

[14] Pearson N Pradeilles R Kingsnorth A Peral-Suarez A Boxer B Griffiths P . The effectiveness of combined dietary and physical activity interventions for improving dietary behaviors, physical activity, and adiposity outcomes in adolescents globally: a systematic review and meta-analysis. Obes Rev. (2025) 26:e13940. doi: 10.1111/obr.13940 40393933 PMC12318910

[15] Allcott-Watson H Chater A Howlett N Troop N. A systematic review of interventions targeting physical activity and/or healthy eating behaviours in adolescents: practice and training. Health Psychol Rev. (2024) 18:117–40. doi: 10.1080/17437199.2023.2173631, 36722423

[16] Biddle SJH Ciaccioni S Thomas G Vergeer I. Physical activity and mental health in children and adolescents: an updated review of reviews and an analysis of causality. Psychol Sport Exerc. (2019) 42:146–55. doi: 10.1016/j.psychsport.2018.08.011

[17] Rodriguez-Ayllon M Cadenas-Sanchez C Estévez-López F Muñoz NE Mora-Gonzalez J Migueles JH . Role of physical activity and sedentary behavior in the mental health of preschoolers, children and adolescents: a systematic review and meta-analysis. Sports Med. (2019) 49:1383–410. doi: 10.1007/s40279-019-01099-5, 30993594

[18] Bourke M Hilland TA Craike M. A systematic review of the within-person association between physical activity and affect in children’s and adolescents’ daily lives. Psychol Sport Exerc. (2021) 52:101825. doi: 10.1016/j.psychsport.2020.101825

[19] O’Neil A Quirk SE Housden S Brennan SL Williams LJ Pasco JA . Relationship between diet and mental health in children and adolescents: a systematic review. Am J Public Health. (2014) 104:e31–42. doi: 10.2105/AJPH.2014.302110, 25208008 PMC4167107

[20] Wang Y Liu B Hu Y Li X Zhang L. Associations between dietary intake, diet quality and depressive symptoms in youth: a systematic review of observational studies. Public Health Nutr. (2022) 26:285–99. doi: 10.1017/S1368980022002656 PMC9808911 36686054

[21] Raghuveer G Hartz J Lubans DR Takken T Wiltz JL Mietus-Snyder M . Cardiorespiratory fitness in youth: an important marker of health: a scientific statement from the American Heart Association. Circulation. (2020) 142:e101–18. doi: 10.1161/CIR.0000000000000866, 32686505 PMC7524041

[22] World Health Organization (2026) Global school-based student health survey. Available online at: https://extranet.who.int/ncdsmicrodata/index.php/catalog/GSHS (Accessed June 1, 2026)

[23] Beal T Morris SS Tumilowicz A. Global patterns of adolescent fruit, vegetable, carbonated soft drink, and fast-food consumption: a meta-analysis of global school-based student health surveys. Food Nutr Bull. (2019) 40:444–59. doi: 10.1177/0379572119848287, 31617415

[24] Bollen KA Bauldry S. Three Cs in measurement models: causal indicators, composite indicators, and covariates. Psychol Methods. (2011) 16:265–84. doi: 10.1037/a0024448, 21767021 PMC3889475

[25] Kowalski KC Crocker PRE Kowalski NP. Convergent validity of the physical activity questionnaire for adolescents. Pediatr Exerc Sci. (1997) 9:342–52. doi: 10.1123/pes.9.4.342

[26] Kowalski KC Crocker PRE Donen RM. The Physical Activity Questionnaire for Older Children (PAQ-C) and Adolescents (PAQ-A) Manual. Saskatoon: College of Kinesiology, University of Saskatchewan (2004).

[27] Li X Wang Y Li X Li D Sun C Xie M . Revision of the Chinese version of the physical activity questionnaire for adolescents (PAQ-A) and evaluation of its reliability and validity. J Beijing Sport Univ. (2015) 38:63–7. doi: 10.19582/j.cnki.11-3785/g8.2015.05.012

[28] Ekkekakis P Zenko Z Vazou S. Do you find exercise pleasant or unpleasant? The affective exercise experiences (AFFEXX) questionnaire. Psychol Sport Exerc. (2021) 55:101930. doi: 10.1016/j.psychsport.2021.101930

[29] Ortega FB Ruiz JR Castillo MJ Sjöström M. Physical fitness in childhood and adolescence: a powerful marker of health. Int J Obes. (2008) 32:1–11. doi: 10.1038/sj.ijo.0803774, 18043605

[30] McNeish D. Thanks coefficient alpha, we’ll take it from here. Psychol Methods. (2018) 23:412–33. doi: 10.1037/met0000144, 28557467

[31] Putnick DL Bornstein MH. Measurement invariance conventions and reporting: the state of the art and future directions for psychological research. Dev Rev. (2016) 41:71–90. doi: 10.1016/j.dr.2016.06.004, 27942093 PMC5145197

[32] Hu LT Bentler PM. Cutoff criteria for fit indexes in covariance structure analysis: conventional criteria versus new alternatives. Struct Equ Model. (1999) 6:1–55. doi: 10.1080/10705519909540118

[33] Chen FF. Sensitivity of goodness of fit indexes to lack of measurement invariance. Struct Equ Model Multidiscip J. (2007) 14:464–504. doi: 10.1080/10705510701301834

[34] Cheung GW Rensvold RB. Evaluating goodness-of-fit indexes for testing measurement invariance. Struct Equ Model Multidiscip J. (2002) 9:233–55. doi: 10.1207/S15328007SEM0902_5

[35] Benjamini Y Hochberg Y. Controlling the false discovery rate: a practical and powerful approach to multiple testing. J R Stat Soc Ser B Stat Methodol. (1995) 57:289–300. doi: 10.1111/j.2517-6161.1995.tb02031.x

[36] World Health Organization and United Nations Educational, Scientific and Cultural Organization. Making Every School a Health-Promoting School: Global Standards and Indicators. Geneva: World Health Organization and UNESCO (2021).

