# Supplementary material for: Dynamic associations among selected healthy dietary behaviors, physical activity, emotion, and fitness performance in adolescents: a four-wave random intercept cross-lagged panel study
Source: Front Nutr. 2026 Aug 3;13:1900491. doi: 10.3389/fnut.2026.1900491 (PMC13477904; doi:10.3389/fnut.2026.1900491)
Supplement: Supplementary file 1 [file Table_1.DOCX]

**Supplementary Material**

Supplementary tables for measurement information

**Supplementary Table S1. Item wording and response options for selected dietary behavior and emotion measures**

| **Construct** | **Indicator / item** | **English item wording** | **Response options** | **Scoring in the present study** |
| --- | --- | --- | --- | --- |
| Selected healthy dietary behaviors | Fruit intake | During the past 7 days, on how many days did you eat fruit? | 1 day; 2–3 days; 4 days; 5 days; 6–7 days | Original direction; higher scores indicate more frequent fruit intake. |
| Selected healthy dietary behaviors | Vegetable intake | During the past 7 days, on how many days did you eat vegetables? | 1 day; 2–3 days; 4 days; 5 days; 6–7 days | Original direction; higher scores indicate more frequent vegetable intake. |
| Selected healthy dietary behaviors | Sugar-sweetened beverage consumption | During the past 7 days, on how many days did you drink sugar-sweetened beverages? | 1 day; 2–3 days; 4 days; 5 days; 6–7 days | Reverse-coded; higher recoded scores indicate lower sugar-sweetened beverage consumption. |
| Selected healthy dietary behaviors | Fast-food consumption | During the past 7 days, on how many days did you eat fast food? | 1 day; 2–3 days; 4 days; 5 days; 6–7 days | Reverse-coded; higher recoded scores indicate lower fast-food consumption. |
| Emotion | Pleasure–displeasure | During physical activity in the past 7 days, I felt pleasant rather than unpleasant. | Five-point scale, from very unpleasant to very pleasant | Higher scores indicate a better emotional state. |
| Emotion | Energy–tiredness | During physical activity in the past 7 days, I felt energetic rather than tired. | Five-point scale, from very tired to very energetic | Higher scores indicate a better emotional state. |
| Emotion | Calmness–tension | During physical activity in the past 7 days, I felt calm rather than tense. | Five-point scale, from very tense to very calm | Higher scores indicate a better emotional state. |

**Note.** The dietary items were adapted from the Global School-based Student Health Survey framework and were scored on a five-point past-7-day frequency scale. Fruit and vegetable intake were coded in the original direction, whereas sugar-sweetened beverage and fast-food consumption were reverse-coded. The emotion items were adapted from the core affective exercise experiences component of the AFFEXX questionnaire. The English wording is provided as a translation of the Chinese survey items.

**Supplementary Table S2. Standardized factor loadings for the three emotion items across four waves**

| **Emotion item** | **T1 λ (SE)** | **T2 λ (SE)** | **T3 λ (SE)** | **T4 λ (SE)** |
| --- | --- | --- | --- | --- |
| Pleasure–displeasure | .794 (.008) | .760 (.004) | .728 (.006) | .764 (.007) |
| Energy–tiredness | .771 (.008) | .737 (.003) | .773 (.006) | .739 (.007) |
| Calmness–tension | .786 (.008) | .719 (.003) | .745 (.006) | .771 (.007) |

**Note.** Values are STDYX standardized factor loadings from the scalar longitudinal measurement invariance model for the emotion construct. All factor loadings were statistically significant at p < .001. λ = standardized factor loading; SE = standard error.
